# A new, effective method for diagnosing GLAD lesions: the chicken-wing muscle up test

**DOI:** 10.1186/s12891-024-07699-1

**Published:** 2024-07-30

**Authors:** Shun Lin, Zhenye Zhong, Jie Xiao, Yun Zheng, Feng Shen, Jinshui Chen, Xiu Yang, Xuesong Han

**Affiliations:** 1 Department of Orthopedics, Fuzong Clinical Medical College of Fujian Medical University, Fuzhou, 350025 China; 2 Department of Orthopedics, the 900th Hospital of Joint Logistic Support Force, PLA, Fuzhou, 350025 China; 3 Department of Orthopedics, Fuzhou Second Hospital, Fuzhou, 350007 China

**Keywords:** Glenoid labral lesions, GLAD lesions, Chicken-wing muscle up test, Shoulder

## Abstract

**Purpose:**

We aimed to develop and evaluate a new diagnostic method, the 'chicken-wing muscle up test', to improve the accuracy of diagnosis of glenolabral articular disruption (GLAD) lesions compared to currently used clinical tests for injuries to the labrum.

**Methods:**

Preoperative evaluations were conducted on 85 patients undergoing arthroscopic surgery at a single center between July 2021 to July 2022. The diagnostic performance of the preoperative clinical examinations (chicken-wing muscle up test, O'Brien test, crank test, and O'Driscoll test) were validated against the findings of arthroscopic examinations.

**Results:**

12 of the 85 patients in this study had arthroscopically confirmed GLAD lesions. The chicken-wing muscle up test demonstrated significantly higher sensitivity (83.33%) for GLAD lesions than the O'Brien test (33.33%), but not the crank test (50.00%) or O'Driscoll test (25.00%), and significantly higher specificity (95.89%) than the O'Brien test (75.34%), crank test (82.19%), and O'Driscoll test (71.23%). The chicken-wing muscle up test had the largest area under the receiver operating characteristic curve (AUC = 0.896, *P* < 0.001; O'Driscoll test AUC = 0.543, *P* > 0.05; crank test AUC = 0.661, *P* > 0.05; O'Brien test AUC = 0.481, *P* > 0.05), indicating significantly better diagnostic efficacy for GLAD lesions than the other three tests.

**Conclusions:**

The chicken-wing muscle up test is a reliable diagnostic method that improves the accuracy of diagnosis of GLAD lesions.

Glenolabral articular disruption (GLAD) lesions in the anterior inferior glenoid labral-cartilage complex were first arthroscopically identified and proposed by Neviaserin 1993 [1]. The lesions are characterized as a nondisplaced tear of the anterior inferior glenoid labrum of the shoulder with adjacent cartilage damage. The glenoid labrum tear remains attached to the periosteum of the scapular glenoid, and the injuries often involve characteristic flaps of cartilage or free bodies. The mechanism leading to these lesions is thought to be related to sudden adduction of the shoulder joint during abduction and external rotation. The main symptom is persistent pain with a popping sound in the anterior aspect of the shoulder; however, joint instability is absent. Due to the unusual symptoms and absence of clear positive signs for GALD lesions during physical examination, coupled with the low incidence of this injury (reportedly only 1.5‒2.9% [2–4]), patients can overlook and inexperienced physicians may misdiagnose this injury, leading to delayed treatment.

The most commonly used clinical examinations for injuries to the labrum are the O'Brien test [5], the crank test [6], and the O'Driscoll test [7]. However, these tests may not be sensitive for detection of GLAD lesions. At our clinic, we observed that many athletic patients with GLAD lesions were hurt while performing a movement called the 'chicken-wing muscle up', and then became unable to perform this movement again. In this pull-up movement on a bar, the upper arm on one side (usually the dominant side) is abducted and externally rotated at the shoulder to perform internal rotation, enabling the shoulder joint on this side of the body to reach the top of the bar. Then, the application of force allows the other side of the shoulder joint to cross and lift the upper body above the bar. We hypothesize that injuries leading to new inability to complete this movement are closely associated with GLAD lesions. Therefore, in this study, we designed, proposed, and evaluated a new clinical investigation test called the 'chicken-wing muscle up test' with the goal of improving the diagnosis of GLAD lesions.

## Patients and methods

In this retrospective clinical case study, all patients provided written consent for treatment and surgery. All patients undergoing shoulder arthroscopy at the 900th Hospital of Joint Logistics Support Force between July 2021 and July 2022 were eligible for inclusion. Patients with a history of shoulder dislocation, previous shoulder surgery, or shoulder stiffness requiring release of adhesions were excluded from the study group. After excluding these patients, the study group comprised 85 patients with shoulder injuries. All patients underwent a detailed preoperative history, which mainly included gender, age, dominant side, a history of trauma, VAS score, and preoperative X-rays and MRI were perfected. All patients were examined by the same senior surgeon, with the physical examinations including the chicken-wing muscle up test, O'Brien test, crank test, and O'Driscoll test.

### Chicken-wing muscle up test

**Fig. 1:**
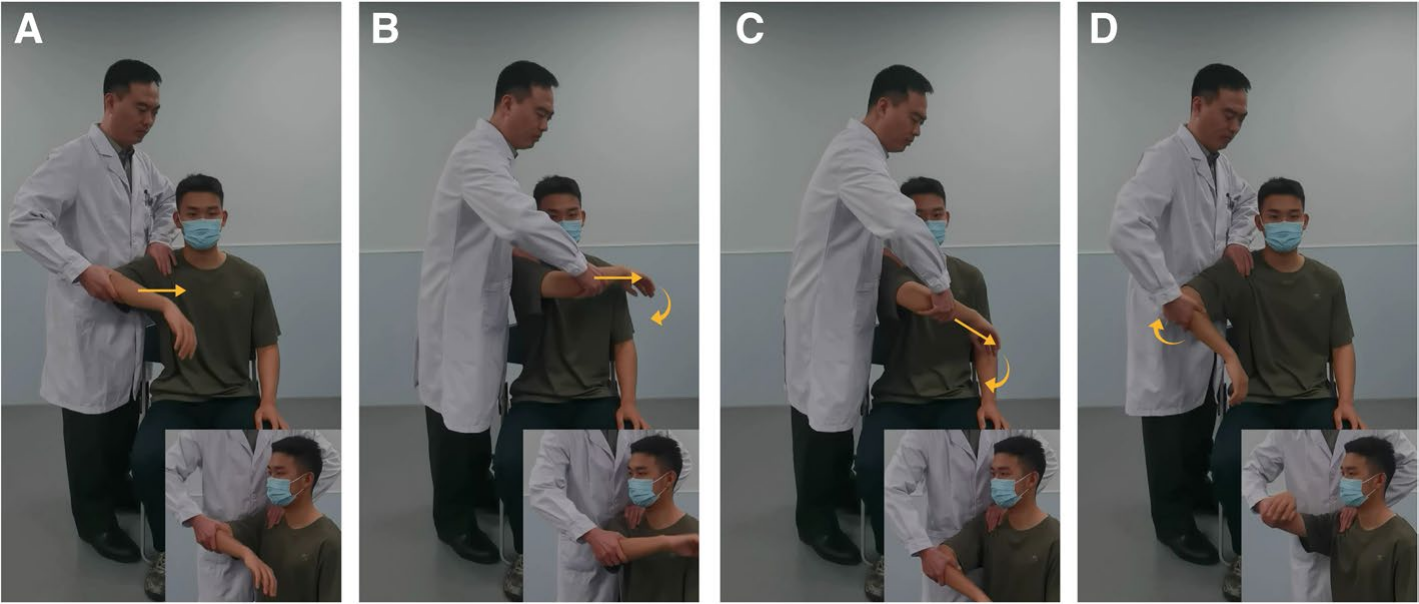
Images of the 'chicken-wing muscle up test'. **A** The examiner places the patient’s affected shoulder in a neutral position with the forearm in 90° abduction, holding the patient’s elbow with one hand and performing shoulder adduction (the index and middle fingers of the examiner’s other hand are placed over the position of the anterior inferior glenoid rim of the articular glenoid at the 3‒5 o'clock position throughout, squeezing the anterior inferior glenoid rim). **B**-**C** While maintaining internal retraction of the shoulder joint, the examiner continues with internal rotation and applies downward pressure on the humeral head, and should feel impingement and compression of the humeral head on the anterior inferior glenoid rim. **D** The examiner gradually abducts the patient’s shoulder while maintaining internal rotation, returning the shoulder to the neutral abduction position to repeat the test

The examiner should stand on the patient’s affected side and lay the part of their hand between the thumb and the index finger on the outer edge of the patient's acromion. The patient’s scapula is fixed with the examiner’s thumb and ring finger to prevent rotation and movement. The examiner places the index and middle fingers of their other hand on the patient's anterior-inferior glenoid rim, specifically at the three to five of o'clock position of the patient's glenoid. The examiner should then apply pressure to the soft tissues of the anterior-inferior glenoid rim in the direction of the humeral head, and observe whether any relative movement occurs and listen for a popping sound between the humeral head and the glenoid (Fig. 1A). Holding the patient’s elbow of the patient with their other hand, the examiner should gradually adduct the shoulder joint, internally rotate the shoulder joint, and press down the upper arm from a neutral or slightly externally rotated position in shoulder abduction of 90° ~ 120°. This movement causes the humeral head to produce an impacting and grinding force on the glenoid labrum and the cartilage of the anterior inferior glenoid rim (Fig. 1B‒C). Then, the examiner should return the patient’s shoulder to the initial position (Fig. 1D) and repeat the movement several times. The examiner should be able to feel the humeral head pressing against the anterior inferior glenoid rim using their index and middle fingers. It is considered a positive sign if the patient reports pain in the anterior part of the shoulder, with or without a popping sound.

### O'Brien test

The O’Brien test was performed as previously described [7]. Briefly, the patient assumes a standing position with the affected shoulder flexed 90° forward and 15° inward. The upper arm is then internally rotated to resist the downward force and externally rotated to resist the downward force. A positive test is indicated by pain in the anterior aspect of the shoulder joint during the internally rotated position of the upper arm and pain relief when the upper arm is in the externally rotated position.

### Crank test

The examiner abducts the patient's arm to 160°. While steadying the patient’s scapula with one hand, the examiner applies controlled rotational force to the glenohumeral joint with the other hand, achieving maximal internal and external rotation. Then, while still holding the scapula, the examiner loads the glenohumeral joint towards the shaft and rotates the arm maximally inwards and outwards. A positive outcome is determined by the presence of pain, either accompanied or unaccompanied by an audible clicking sound, specifically observed during external rotation [8].

### O'Driscoll test

The patient is positioned either seated or supine, with their arm placed laterally and the elbow bend at 90°. The arm is externally rotated to 90° and then abducted to 90°. The examiner proceeds to flex the elbow and further raise the arm to 120°. A positive test result is characterized by the reproduction of pain during 90° to 120° abduction, often accompanied by a distressing clicking sound within the 90° to 120° range [9].

### Arthroscopy

**Fig. 2:**
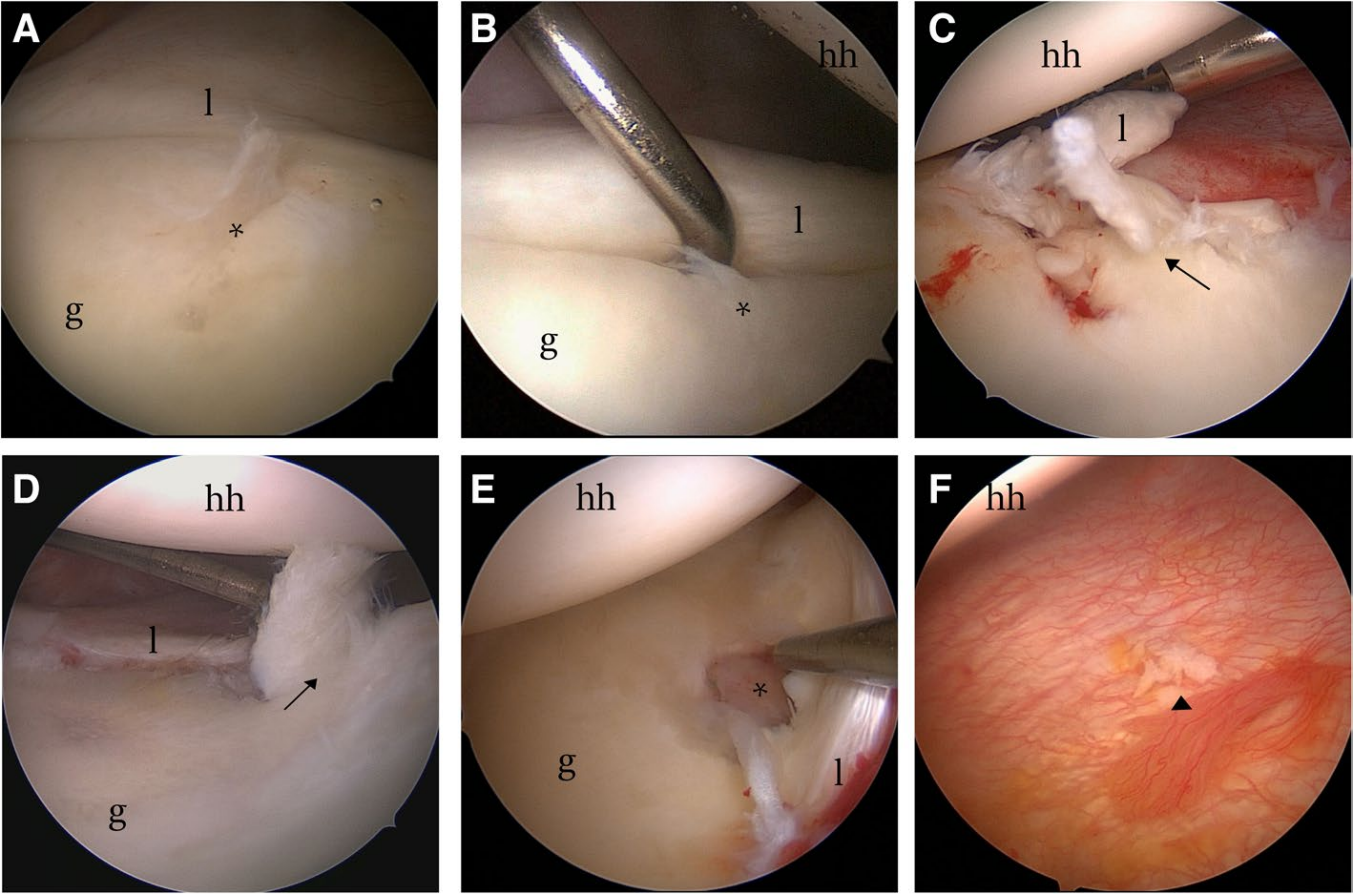
Arthroscopic presentation of GLAD lesions. **A**-**B** A mild injury to the anterior inferior glenoid labrum combined with slight damage to the cartilage. **C**-**D** Flap-like lifting of the cartilage is observed, and both the cartilage and the glenoid lip are torn, separating from the glenoid rim. **E** Injury to the anterior labrum of the lower glenoid is accompanied by severe cartilage damage and exposure of the subchondral bone. **G** Cartilage fragments forming intra-articular free bodies. hh indicates the humeral head, g indicates the glenoid, l indicates the labrum, * indicates the damaged cartilage surface, black arrows indicate the damaged cartilage flaps, and ▲ indicates the free body formed by a detached cartilage flap

After general anesthesia via tracheal intubation, the patients were placed on the contralateral side of the affected limb with their head suitably elevated and immobilized, with the tension of the neck and shoulder adjusted to prevent injury to the brachial plexus nerve. To ensure adequate space for surgical manipulation, a 6 kg weight was used to suspend the affected limb and open the joint cavity. An exploration channel was created from the back of the shoulder capsule under the ridge of the scapula, and two working channels were established anteriorly under direct vision (if further treatment was required). An arthroscope with a wide-angle lens was used to sequentially examine the structures inside the shoulder joint to confirm the absence of injuries elsewhere. The position of the anterior inferior glenoid rim was then exposed for observation as the arm was manipulated in abduction and external rotation. The presence of any of the following findings on observing labrum and cartilage injuries indicates GLAD lesions: (1) type I: an anterior inferior glenoid labral injury combined with mild nearby cartilage damage, but no cartilage flap or cartilage defect (Fig. 2A-B); (2) type II: the glenoid labrum near the anterior inferior glenoid rim has broken off, typically with tearing of the cartilaginous flap together with the glenoid labrum (Fig. 2C‒D); and (3) type III: the tip of the cartilage flap has broken off but has detached and formed an intra-articular free body or been ground into tiny cartilage fragments (Fig. 2E); a typical 'cartilage flap' is not visible. In this case, there is a break of the anterior inferior labrum, along with cartilage defects near the anterior inferior glenoid rim, and free bodies formed by cartilage fragments may be found in the capsule (Fig. 2F).

### Data processing

Diagnostic arthroscopy is widely recognized as the most reliable method for diagnosing GLAD lesions. We calculated the number of true-positive, true-negative, false-positive, and false-negative results for each of the four diagnostic methods to determine their sensitivity, specificity, positive predictive value, negative predictive value, and accuracy. McNemar's tests were used to compare the sensitivity and specificity of these methods. Additionally, we constructed receiver operating characteristic (ROC) curves and calculated the areas under the curve (AUC) to evaluate the diagnostic performance of each test for detecting GLAD lesions. Statistical significance was considered at the *P* < 0.05 level. All statistical analyses were performed using IBM SPSS Statistics version 26.0.

## Results

A total of 85 individuals with injuries of the shoulder were included in this study. Diagnostic arthroscopy revealed 34 glenoid labral injuries, including 12 GLAD lesion (6 type I, 3 type II and 3 type III), 5 Bankart lesions, 1 total labral lesions, 1 reverse Bankart lesions, and 15 SLAP injuries, 47 rotator cuff tears, 2 rotator cuff tears with bicep tendon injuries, and 2 rotator cuff tears with labrum injuries (Table 1).

**Table 1:** Demographic data of the studied group

No. of patients	85
Age, years (range)	42.95 ± 16.19 (20–81)
Sex	
Male	49
Female	36
Dominance	
Dominant	59
Nondominant	26
Type of injury	
GLAD lesions	12
Bankart lesions	5
Reverse Bankart lesions	1
SLAP injuries	15
Total labral lesions	1
Rotator cuff tears	47
Rotator cuff tears with bicep tendon injuries	2
Rotator cuff tears with labrum injuries	2
VAS score (0–10)	6.26 ± 1.23
History of trauma	71

*VAS* Visual analog scale for pain. Continuous data are expressed as mean ± standard deviation

The prevalence of GLAD lesions in this study was approximately 14.12%. The chicken-wing muscle up test missed 2 type I GLAD lesions, resulting in false negatives. In contrast, the crank test missed 8, the O'Brien test missed 9, and the O'Driscoll test missed 6. The chicken-wing muscle up test produced three false positives (3 cases with the following pathology found on arthroscopy: 2 Bankart lesions, 1 SLAP injuries). The O’Driscoll test had 18 false positive, the crank test had 13 false positive, and the O 'Brien test yielded 21 false positives for GALD lesions (Table 2).

**Table 2:** Diagnostic performance of the four clinical tests for GLAD lesions

	Chicken-wing muscle up test	O ‘Driscoll test	Crank test	O’Brien test
True-Positive	10	4	6	3
True-Negative	70	55	60	52
False-Positive	3	18	13	21
False-Negative	2	8	6	9
Positive Predictive Value	76.92%	18.18%	31. 58%	12.50%
Negative Predictive Value	97.22%	87.30%	90. 91%	85. 25%
Sensitivity	83.33%	33.33%	50.00%	25.00%
Specificity	95.89%	75.34%	82.19%	71.23%
Accuracy	94.12%	69.41%	77. 65%	64. 71%
AUC	0. 896	0.543	0.661	0.519

### Sensitivity and specificity

The sensitivity of the chicken-wing muscle up test for GLAD lesions was 83.33% compared to 33.33% for the O’Driscoll test, 50.00% for the crank test, and 25.00% for the O'Brien test. The chicken-wing muscle up test demonstrated significantly higher sensitivity for GLAD lesions than the O'Brien test (*P* = 0.039), but not the O'Driscoll test (*P* = 0.070) or crank test (*P* = 0.219). On the basis of these results, power analyses revealed that increasing the sample size by 140% would detect at least 28 GLAD lesions, enabling a Fisher’s test with 80% power at the 0.05 significance level to distinguish between the sensitivity of the chicken-wing muscle up test and both the O'Driscoll test and crank test.

The specificity of the chicken-wing muscle up test for GLAD lesions was 95.89%, compared to 75.34% for the O’Driscoll test, 82.19% for the crank test, and 71.23% for the O'Brien test. The chicken-wing muscle up test had significantly higher specificity than the O’Driscoll test (*P* < 0.001), the crank test (*P* < 0.05), and the O'Brien test (*P* < 0.001).

### Positive predictive value and negative predictive value

The chicken-wing muscle up test had a positive predictive value for GLAD lesions of 76. 92% compared to 18.18% for the O’Driscoll test, 31.58% for the crank test, and 12.50% for the O'Brien test.

The negative predictive value of the chicken-wing muscle up test for GLAD lesions was 97.22% versus to 87.30% for the O’Driscoll test, 90.91% for the crank test, 85.25% for the O'Brien test.

### Accuracy

The accuracy of the chicken-wing muscle up test for GLAD lesions was 94.12% compared to 69.41% for the O’Driscoll test, 77.65% for the crank test, and 64.71% for the O'Brien test.

### ROC curves and area under the curve

**Fig. 3:**
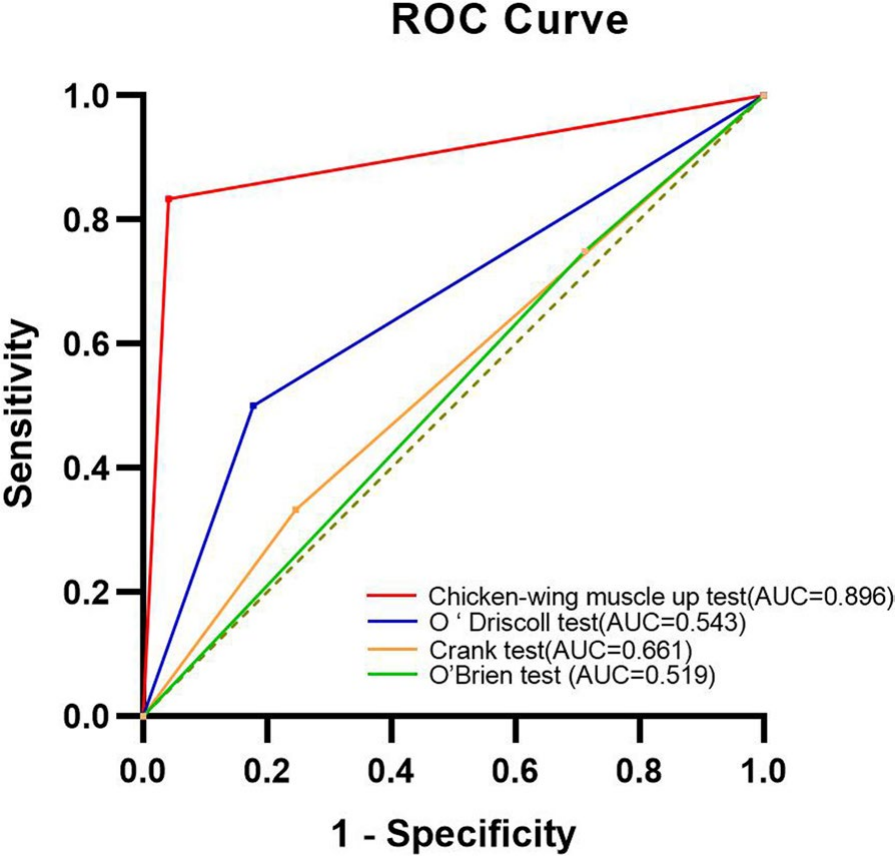
ROC curve analysis of four clinical tests to diagnose GLAD lesions

The AUC was 0.896 (*P* < 0.001) for chicken-wing muscle up test, 0.543 (*P* > 0.05) for the O’Driscoll test, 0. 661 (*P* > 0.05) for the crank test, and 0. 481 (*P* > 0.05) for the O'Brien test (Fig. 3).

## Discussion

This study included 85 patients with with shoulder injuries that required surgery, 12 of whom had arthroscopically confirmed GLAD lesions. This indicates that the incidence of GLAD lesions is not low, in contrast to previous reports [2, 3]. However, the rate of detection of GLAD lesions is low. Firstly, there is a lack of suitable clinical tests for GLAD lesions. Our results indicate that conventional labral clinical tests, such as the O'Driscoll test, the crank test, and the O'Brien test, have sensitivities of no more than 50% for GLAD lesions. Therefore, accurate preoperative diagnosis cannot be made solely based on conventional clinical tests. Secondly, the preoperative diagnosis of GLAD is highly dependent on MRI arthrography, as the diagnostic value of CT and conventional MRI for these lesions are low [1]. One study reported that MRI arthrography is 100% accurate [8]; however, other studies showed that the sensitivity of MRI arthrography for glenoid chondromalacia is only 65% and 67% [9] with significant inter- and intraobserver variability observed. At present, it is difficult to accurately diagnose GLAD lesions and effectively contribute to surgical planning based on preoperative MRI and imaging results alone. Preoperative evaluation of GLAD lesions is challenging due to the low sensitivity of MRI and the cost and invasiveness of MRI arthrography. Therefore, more accurate assessment tools are necessary to avoid missed and untreated GLAD lesions, as conservative treatment is unsatisfactory and special intraoperative devices and implants are required for treatment.

**Fig. 4:**
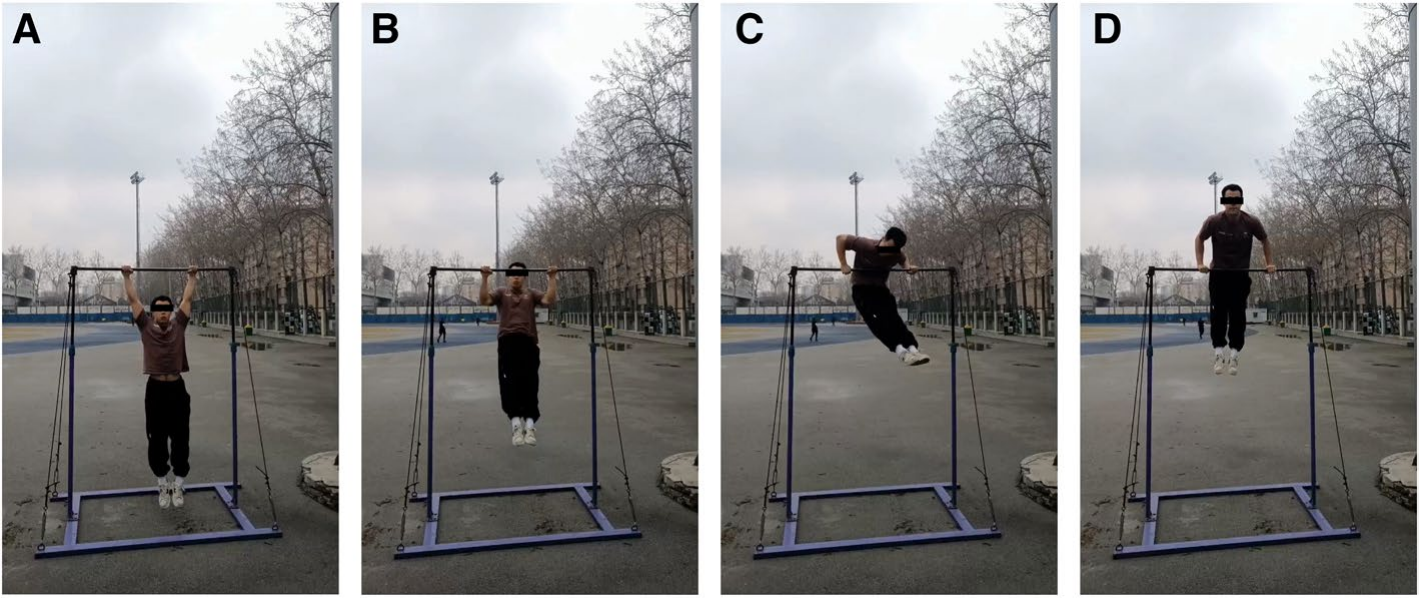
Photographs of a individual performing the chicken-wing muscle up movement. **A**-**B** Abduction of the shoulder joints bilaterally and elevation of the body. **C** The dominant shoulder joint performs internal rotation and adduction from an abducted external rotation position, causing one side of the upper body to rise above the bar. **D** The dominant shoulder joint assists with the adduction and internal rotation of the other shoulder joint to elevate the upper body above the bar

It is noteworthy that the prevalence of GLAD lesions is not low in certain populations, particularly in athletes involved in high-intensity motion of the upper extremities [1, 10]. In this study, 11 of the GLAD lesions occurred in athletes involved in high-intensity upper extremity actions. Prior to developing the test proposed in this study, we noted that some patients with GLAD lesions previously treated at our center were injured while performing activities similar to the chicken-wing muscle up movement. Investigation of the movement status of the shoulder joint and forearm at the time the lesions occurred revealed that these patients shared a common mechanism of injury. Specifically, when the dominant side was the first side to exert force to perform shoulder joint adduction and internal rotation to guide the body upward, the shoulder joint of the dominant side became sprained due to insufficient unilateral arm strength and poor control of the core muscles during the body's swing (Fig. 4).

**Fig. 5:**
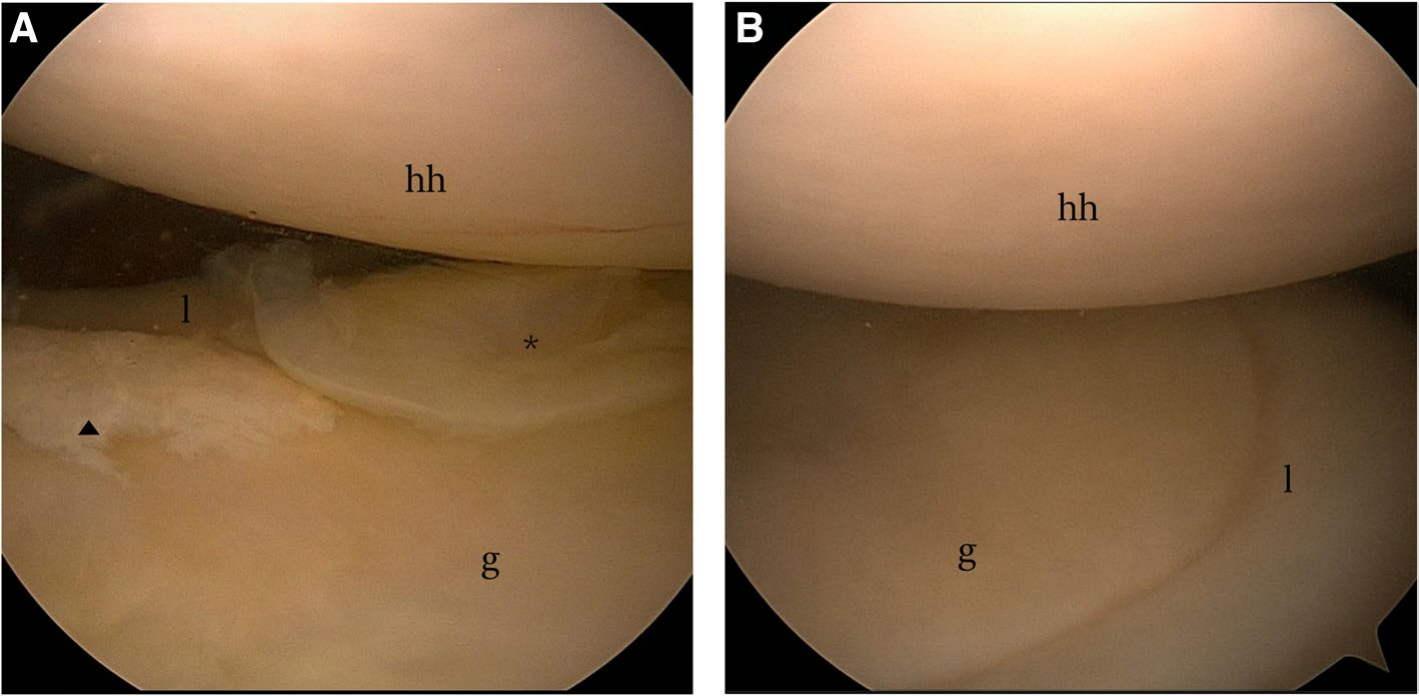
Arthroscopic view during the chicken-wing muscle up test. **A** With the shoulder in the abducting position, there is no significant contact between the humeral head and the anterior or inferior aspect of the glenoid. **B** When adducted, internally rotated, and pressed downwards, the humeral head collides and grinds against the glenoid labrum and cartilage of the anterior inferior glenoid rim. hh indicates the humeral head, g indicates the glenoid, l indicates the labrum, * indicates the damaged cartilage surface, and ▲ indicates the free body

The majority of patients reported hearing a popping sound during their injury, which may suggest a labral or cartilage rupture. This is similar to the mechanism of injury described in the definition of GLAD lesions, in which the shoulder suddenly adducts while in abduction and external rotation [1]. The contusion of the humeral head against the articular cartilage may be exacerbated by internal rotational force. The examination for GLAD lesions proposed in this study simulates the same 'chicken-wing muscle up' movement that caused the injury, by replicating the shoulder movements of an athlete injured during the chicken-wing muscle up movement on a bar. After confirming repeatedly that this maneuver reproduced shoulder pain in other patients who were injured via similar mechanisms, we conducted a this study. In addition, we replicated the chicken-wing muscle up test during arthroscopy and observed anomalous mechanics in the anterior inferior region of the scapular glenoid in patients with GLAD lesions (Fig. 5).

During arthroscopy, the scapula is immobilized and the soft tissues of the anterior inferior glenoid rim are compressed towards the humeral head, then the shoulder joint is gradually adducted, internally rotated, and depressed from a neutral or slightly externally rotated position of the forearm in abduction of 90° to 120. The humeral head applies a shearing force on the glenoid labrum and cartilage of the anterior inferior glenoid rim. This can cause impingement, grinding, and internal mechanical disturbance and displacement, which may result in pain and a clicking sound.

**Fig. 6:**
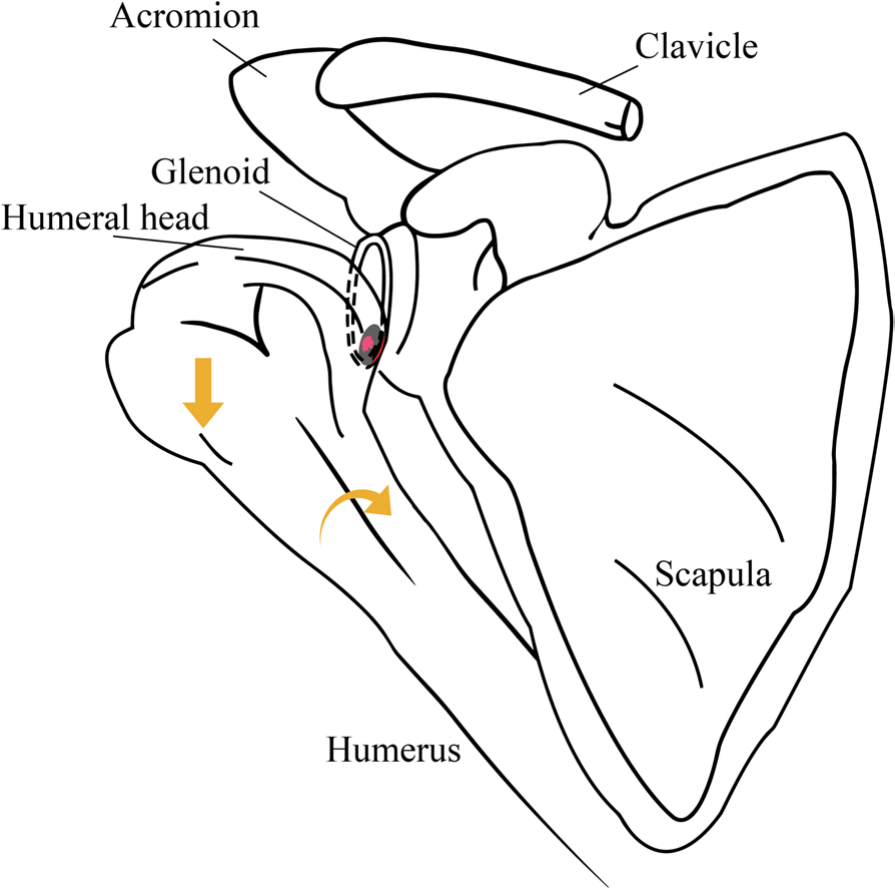
The anatomic basis of the 'chicken-wing muscle up test’. When the arm is internally rotated and internally retracted, and the humeral head is pressed down, the humeral head strikes and grinds the anterior inferior aspect of the scapular glenoid

In this study of 12 patients with GLAD lesions, the chicken-wing muscle up test demonstrated good accuracy in diagnosing GLAD lesions, with a sensitivity of 83.33%, specificity of 95.89%, positive predictive value of 76.92%, negative predictive value of 97.22%, accuracy of 94.12%, and AUC of 0.896 (*P* < 0.001). The overall diagnostic performance of the chicken-wing muscle up test was significantly better than the other three clinical tests. While there are certain similarities between Chicken-wing muscle up test and O'Brien test, crank test, and O'Driscoll test, there are also notable differences in the details and mechanisms of the examinations. The O'Brien test, the crank test, and the O'Driscoll test are all mainly used as physical examinations for superior glenoid labral tears of the shoulder. The underlying mechanisms are generally either direct impingement of the raised humeral head with the injured superior glenoid labral, causing labral displacement, or pulling and shearing of the glenoid labrum by the biceps tendon, or both. This results in mechanical disturbances and displacements within the tissues that cause pain in the patient [11]. However, all three tests ignored the anterior inferior glenoid rim. The Chicken-wing muscle up test applies downward pressure on the humeral head, rather than elevation of the humeral head. At the same time, the humeral head is internally retracted and internally rotated to bring the humeral head as close as possible to the anterior inferior aspect of the scapular glenoid. Further internal rotation creates a shearing force on the anterior inferior glenoid labrum, which causes the humeral head to press further against the glenoid, generating a crushing, grinding force (Fig. 6). Joint popping and pain are caused by cartilage damage and glenoid labral tears in the anterior inferior aspect of the glenoid.

If a positive chicken-wing muscle up test is observed, the doctor should pay close attention to cartilage damage in the anterior inferior aspect of the glenoid during arthroscopic evaluation. In this study, the chicken-wing muscle up test failed to detect GLAD lesions in two patients. These two patients with false-negative results had only minor cartilage damage, relatively minor labral tears, and significant displacement was not observed arthroscopically. To avoid this issue, the examination maneuvers of the chicken-wing muscle up test need to be repeated several times. The examiner should squeeze the soft tissues of the anterior inferior glenoid rim towards the humeral head to adequately grind the anterior inferior position of the glenoid, which may reduce the probability of missing the diagnosis.

In addition, the chicken-wing muscle up test produced false positives in 2 cases of Bankart injuries and 1 case of SLAP injuries. Two patients with Bankart injuries had false positives during the chicken-wing muscle up test, one of whom had experienced subluxation. During the chicken-wing muscle up test, the affected shoulder is abducted and slightly externally rotated, similar to the movement of the shoulder apprehension test [12, 13]. It has been found that a mildly unstable shoulder in abduction (between 45° and 80°) and external rotation causes similar pain to that caused by compression of the glenohumeral ligament complex [14]; thus, this false positive is well documented. False positives may also occur when performing the chicken-wing muscle up test in patients with shoulder instability due to pain from dislocation. Therefore, it is important to pay attention to the following techniques when examining patients. The examiner should use one hand to fix the patient’s scapula and use their index, middle, and ring fingers to block the anterior and inferior part of the joint capsule. This will help prevent the shoulder joint from dislocating and reduce the possibility of false positives. The chicken-wing muscle up test may also result in false positives in patients with SLAP injuries. For instance, one patient with a false-positive SLAP injury in the chicken-wing muscle up test was observed arthroscopically to have an injury involving the anterior superior labrum with severe cartilage damage. Patients do not usually report pain when the humeral head compresses the superior glenoid labrum during the chicken-wing muscle up test. However, if the examiner fails to depress the humeral head adequately, the collision with the glenoid labral complex of the biceps longus tendon can cause pain and result in a false-positive chicken-wing muscle up test. It is important to be aware that insufficient depression of the humeral head during the examination may cause pain and result in a false positive by squeezing upwards on the injured rotator cuff. Therefore, it is crucial to ensure that the humeral head is properly depressed to avoid false positives. In these patients, there is no popping sound during the examination, and the pain is mostly located at the rotator cuff insertion [15]. In contrast, patients with GLAD lesions experience pain mostly anterior to the shoulder.

Generally speaking, our results indicate the chicken-wing muscle up test is the most reliable clinical examination for detection of GLAD lesions. A positive result alerts the surgeon to pay special attention to injuries to the anterior inferior aspect of the scapular glenoid during arthroscopic evaluation. Although false positives may occur in some patients with Bankart and SLAP injuries, which affects the positive predictive value of the test, this issue does not diminish the value of the chicken-wing muscle up test in clinical practice. The chicken-wing muscle up test was designed to identify GLAD lesions in patients with atypical clinical symptoms of shoulder pain and to provide timely treatment to prevent further aggravation of cartilage damage due to missed diagnosis and delayed treatment. After excluding Bankart injuries and SLAP injuries with obvious symptoms and signs through physical examination and imaging, patients who are likely to be at risk for the disease (active young adults, people who exercise their upper limbs frequently) can be screened for insidious GLAD lesions using the chicken-wing muscle up test. This approach may help to avoid underdiagnosis of GLAD lesions. In our cohort, the chicken-wing muscle up test had high sensitivity and specificity, and detected almost all patients with GLAD lesions and excluded most patients with other types of glenoid labral injuries. Therefore, the performance of the chicken-up muscle up test meets our clinical needs and is worthy of further research and validation.

### Limitations

The number of patients is relatively small and a larger sample size may provide a more accurate estimate of the accuracy of these examinations. As this study employs a retrospective design, it is subject to a number of inherent limitations. A further limitation of this study is the potential for detection bias, as the surgeon performing diagnostic arthroscopy was not blinded to the findings. This limitation can be mitigated by implementing a standardized examination protocol.

## Data Availability

The data that support the findings of this study are available on request from the corresponding author upon reasonable request.
